# Incidence and predictors of left ventricular thrombus by cardiovascular magnetic resonance in acute ST-segment elevation myocardial infarction treated by primary percutaneous coronary intervention: a meta-analysis

**DOI:** 10.1186/s12968-018-0494-3

**Published:** 2018-11-08

**Authors:** Heerajnarain Bulluck, Mervyn H. H. Chan, Valeria Paradies, Robert L. Yellon, He H. Ho, Mark Y. Chan, Calvin W. L. Chin, Jack W. Tan, Derek J. Hausenloy

**Affiliations:** 1 Norfolk and Norwich University Hospital, Colney Lane, Norwich, NR4 7UY UK; 2 The Hatter Cardiovascular Institute, Institute of Cardiovascular Science, University College London, London, UK; 3 National Heart Research Institute Singapore, National Heart Centre Singapore, Singapore, Singapore; 4 Department of Cardiology, Tan Tock Seng Hospital, Singapore, Singapore; 5 Department of Cardiology, National University Hospital, Singapore, Singapore; 6 National Heart Centre Singapore, Singapore, Singapore; 7 Barts Heart Centre, St Bartholomew’s Hospital, London, UK; 8 The National Institute of Health Research University College London Hospitals Biomedical Research Centre, London, UK; 9 Cardiovascular and Metabolic Disorders Program, Duke-National University of Singapore, Singapore, Singapore; 10 Yong Loo Lin School of Medicine, National University Singapore, Singapore, Singapore

**Keywords:** Left ventricular thrombus, ST-segment elevation myocardial infarction, Cardiovascular magnetic resonance, Primary percutaneous coronary intervention

## Abstract

**Introduction:**

The incidence of left ventricular (LV) thrombus formation in ST-segment elevation myocardial infarction (STEMI) patients in the current era of primary percutaneous coronary intervention (PCI) is not well established. We performed a meta-analysis to assess the actual incidence and predictors of LV thrombus by cardiovascular magnetic resonance (CMR) in STEMI treated by primary PCI.

**Methods:**

We searched MEDLINE and EMBASE databases up to February 2018. We included all studies published as a full-text article, reporting the incidence of LV thrombus by CMR within 1 month following acute STEMI in patients treated by primary PCI. A binary random-effects model was used to estimate the pooled incidence of LV thrombus. The diagnostic performance of transthoracic echocardiography (TTE) as compared with CMR was pooled to obtain the sensitivity and specificity of TTE with CMR as the gold standard. Embolic and bleeding complications of LV thrombus were also evaluated.

**Results:**

Ten studies were included in the meta-analysis. The incidence of LV thrombus by CMR in all-comer STEMI patients (*n* = 2072) was 6.3% with 96% of LV thrombus occurring in those with anterior STEMI (12.2% incidence). When only anterior STEMI with LVEF< 50% were considered (*n* = 447), the incidence of LV thrombus was 19.2%. Compared with CMR, the sensitivity of TTE to detect LV thrombus was 29% with a specificity of 98%. The sensitivity of TTE increased to 70% in those with anterior STEMI and reduced LVEF. LV thrombus resolved in 88% of cases by 3 to 6 months. After 1–2 years follow-up, the embolic complication rate was similar at 1.5% (*P* = 0.25) but the bleeding complication rate was significantly higher (8.8% versus 0.5%, *P* < 0.001) in the LV thrombus group on triple therapy when compared to the no LV thrombus group on dual antiplatelet therapy.

**Conclusion:**

In the primary PCI era, CMR detection of an LV thrombus post-STEMI remains high with incidence of nearly 20% in anterior STEMI with depressed LVEF. Patients with LV thrombus treated by triple therapy had similar embolic complications but higher bleeding complications than those with no LV thrombus treated with dual antiplatelet therapy. A 3 month follow-up CMR scan to guide anticoagulation duration might help mitigate bleeding risk.

## Background

Primary percutaneous coronary intervention (PCI) is the reperfusion strategy of choice for the treatment of ST-segment elevation myocardial infarction (STEMI). Although left ventricular (LV) thrombus formation is a recognized complication in STEMI patients, its incidence in the era of primary PCI is not well established. LV thrombus may lead to embolic complications such as stroke, with devastating consequences. Both the American College of Cardiology/American Heart Association/and European Society of Cardiology guidelines [1, 2] recommend a minimum of 3 to 6 months of anticoagulation (Class IIb and IIa respectively, level of evidence C) with subsequent repeated imaging to guide ongoing anticoagulation.

A meta-analysis recently reported that the ovrall rate of LV thrombus by transthoracic echocardiography (TTE) in the primary PCI for STEMI era was 2.7% with 9.1% for anterior STEMI [3]. However, TTE has a lower sensitivity (35%) for detecting LV thrombus when compared to cardiovascular magnetic resonance (CMR) [4]. However, TTE relies on the morphological identification of LV thrombus, whereas CMR with gadolinium contrast agent can identify LV thrombus based on both its morphology and tissue characteristics [4].

We therefore performed a meta-analysis to assess the incidence and predictors of LV thrombus by CMR in STEMI patients treated by primary PCI in the current era and to evaluate how it may be used in the clinical setting, in conjunction with TTE, to improve LV thrombus detection.

## Methods

This meta-analysis adhered to the Preferred Reporting Items for Systematic Reviews and Meta-Analyses (PRISMA) statement [5] and was performed according to the recommendations specified in the Cochrane Handbook for Systematic Reviews of Interventions [6].

### Eligibility criteria

All studies reporting on the incidence of LV thrombus by CMR in STEMI patients treated by primary PCI were eligible for inclusion. The inclusion criteria were all studies published as a full-text article reporting the CMR incidence of LV thrombus within 1 month following acute STEMI in patients treated by primary PCI. Conference abstracts were excluded.

### Search strategy

We searched Ovid MEDLINE and Ovid EMBASE databases up to February 2018. Furthermore, we screened editorials and references of eligible studies. The following search terms were used: “ventricular”, “apical”, “thrombus”, “magnetic resonance imaging”, “acute myocardial infarction”, “primary percutaneous intervention”.

### Study selection

**Fig. 1:**
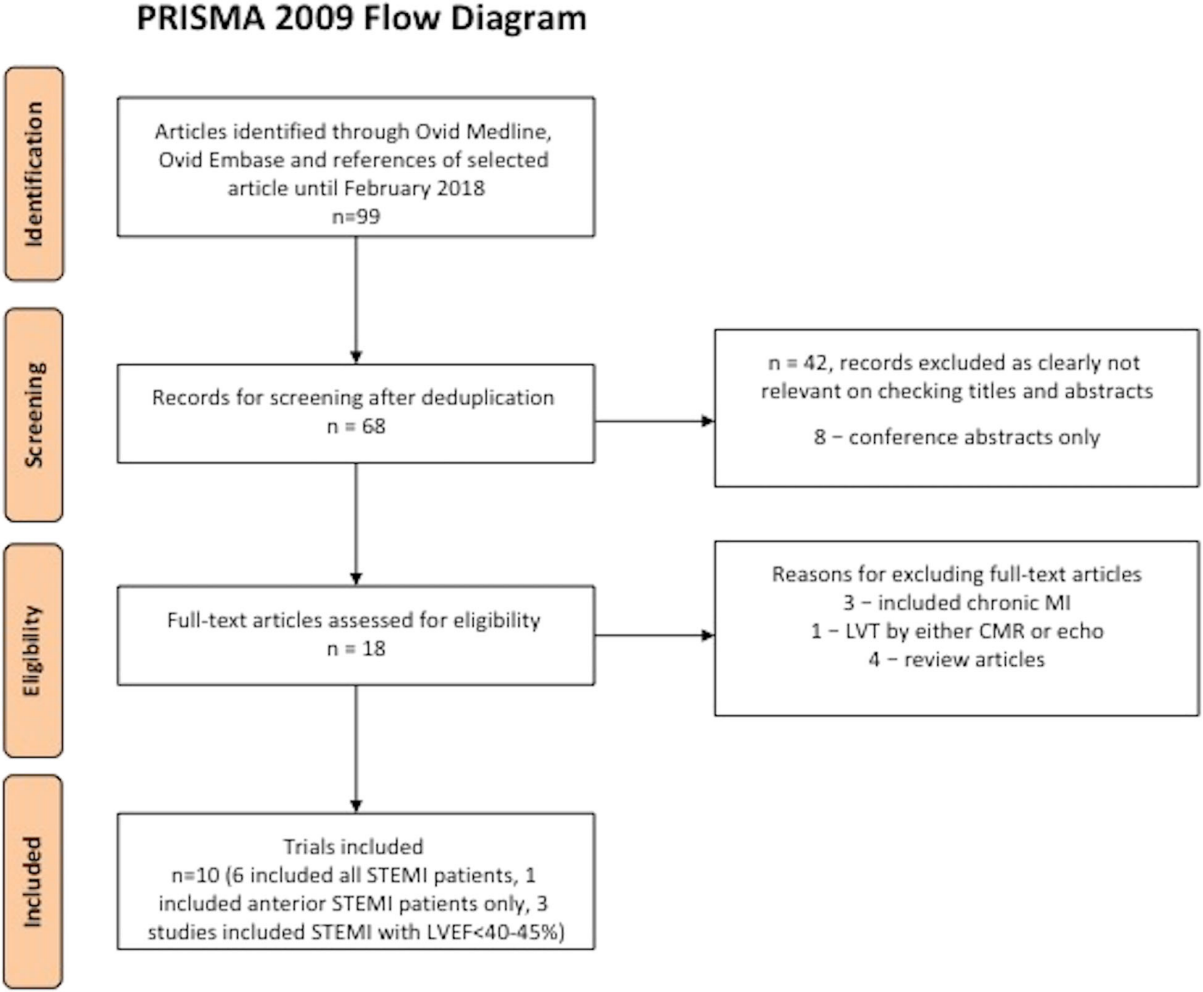
PRISMA flow diagram of the study selection process. This is the PRISMA flow diagram showing how studies were identified, screening and included in this meta-analysis

Two authors (HB, MHHC) identified suitable articles independently. Disagreement was resolved through consensus from a third investigator (DJH). Figure 1 shows the process of study selection as per PRISMA [5].

### Data extraction and quality assessment

Baseline clinical characteristics of the study population were extracted. Trial quality was determined as per the Cochrane Handbook [6] but without constructing a composite quality score given the limitations inherent to such an approach [7]. We aimed to produce a funnel plot if there were > 10 included studies in the forest plots.

### Endpoints

The main endpoints analyzed were incidence of LV thrombus identified on the acute CMR and follow-up CMR when available. Furthermore, the incidences of embolic and bleeding complications as defined by the individual studies were also collected.

### Statistical analysis

The studies were divided into those including all-comer STEMI, anterior STEMI patients and those with anterior STEMI and with reduced LV ejection fraction (LVEF). A binary random-effects model was used to estimate the pooled incidence of LV thrombus with 95% confidence interval (CI) using OpenMeta[Analyst] [8] software. Where available, studies comparing the diagnostic performance of TTE versus CMR were pooled to obtain the sensitivity and specificity of TTE against CMR. Lastly, embolic complications (ischemic stroke and clinically significant peripheral embolisation) and bleeding complications between those with LV thrombus against those without LV thrombus were also compared. Heterogeneity among the studies included in each forest plot was quantified using I^2^ statistics with I^2^ of 0–25%, 25–50% and 50–75% considered as low, moderate and high heterogeneity, respectively.

## Results

Ten studies [4, 9–17] were included in the meta-analysis. The characteristics of these studies are summarized in Table 1. The CMR scan was performed within the first week post-primary PCI in 7 studies [10–16], and between 7 and 30 days in 3 studies [4, 9, 17].

**Table 1:** Characteristics of included studies

Study	Patients	Clinical characteristics	CMR	Technique	Findings	Comments
Delewi 2012 [12]	194 STEMI (123 anterior) Multicentre Substudy of an RCT	Age: 56 ± 9 years Male: 85% DM: 6% Smoker: 52% MI size: 23 ± 12 g	1.5 T Between 2 and 7 (4 ± 2) days and at 4 months	Cine + LGE imaging	17 (8.8%) early LV thrombus and 12 late LV thrombus All LV thrombus were in anterior STEMI	8/17 patients with LV thrombus treated with anticoagulation 15/17 LV thrombus resolved by 4 months
Lanzillo 2013 [11]	36 STEMI (19 anterior) Single centre	Age: 59 ± 10 years Male: 89% DM: 22% Smoker: 69% MI size: 40 ± 13%	1.5 T Within 7 days (5.5 ± 1.4 days)	LGE imaging	7 (19%) LV thrombus All LV thrombus were in anterior STEMI	All resolved by 1 month
Pöss 2015 [10]	738 STEMI (339 anterior) Multicentre Substudy of an RCT	Age: 62 (51–71) years Male: 76% DM: 20% Smoker: 47% MI size: 17 (8–25)%	1.5 T and 3 T CMR between days 1 to 10, median 3 days	Cine + LGE imaging	26/738 (3.5%) LV thrombus 24/26 (92%) we in anterior STEMI	LV thrombus was associated with larger MI size, lower LVEF and increased rate of MACE (Death, re-infarction and new HF)
Weinsaft 2016 [4]	201 STEMI (108 anterior)	Age: 56 ± 12 years Male: 84% DM: 23% Smoker: 32% MI size: 15 (6–23)%	1.5 T Between 7 and 30 days (28 ± 6 days)	Long inversion time LGE	17/201 (8%) LV thrombus 16/17 of the LV thrombus were in anterior STEMI	LV thrombus was associated with apical LV dysfunction
Bière 2016 [14]	329 STEMI (183 anterior) Single centre	Age: 58 ± 11 years Male: 82% DM: 12% Smoker: 43% MI size: 20 ± 13%	1.5 T and 3 T Day 6 (4–8) and at 3 months	First pass perfusion	22 (6.7%) early LV thrombus and 9 (2.9%) late LV thrombus All LV thrombus were in anterior STEMI	FFP improved detection of LV thrombus over cine and LGE images
Cambronero-Cortinas 2017 [13]	574 STEMI patients had the acute CMR only. 392 STEMI (207 anterior) had paired acute and follow-up CMR Single centre	Age: 58 ± 12 years Male: 82% DM: 19% Smoker: 60% MI size: 19 (10–30)%	1.5 T 7 ± 2 days and 6 months (those with LV thrombus were rescanned at 1 year (11/13)	LGE imaging	574 with CMR at 1 week (LV thrombus 28–5%) 18 (5%) early LV thrombus and 9 (2%) late LV thrombus 4/18 (24%) still had LV thrombus at 6 months (anterior) 11 with LV thrombus at 6 months – 3/11 (27%) still had LV thrombus at 1 year (3/25–12%)	LVEF< 50% and anterior STEMI independently predicted LV thrombus (c-statistic 0.82) Patients with anterior infarction and LVEF < 50% are at highest risk of developing LV thrombus (23/115, 20%).
Gellen 2017 [9]	265 anterior STEMI	Age: 58 ± 12 years Male: 85% DM: 44% Smoker: 43% MI size: 31 ± 12%	CMR within 21 days	LGE imaging	34/265 (12.8%) with LV thrombus CMR ≤ 5 days: 13/160 CMR > 5 days: 21/105	The highest LV thrombus detection rate was in patients with CMR performed 9 to 12 days after STEMI
Weir 2009 [15]	100 Acute MI (90 STEMI, 10 NSTEMI) with LVEF< 40%	Age: 59 ± 12 years Male: 77% DM: 0% Smoker: 55% MI size: 33 ± 21 ml/m^2^	CMR at a mean of 4.2 days (range 2–11 days)	First-pass perfusion + LGE	15/100 (15%) with LV thrombus All anterior MI (15/55, 27.3%)	All patients with LV thrombus were formally anticoagulated. No patients with thromboembolic events at 6 months
Surder 2015 [16]	Substudy of SWISS-AMI study 177 anterior STEMI with LVEF< 45%	Age: 57 ± 10 years Male: 85% DM: 11% Smoker: 59% MI size: 29 ± 12%	CMR at a median of 6 (4–8)days	Cine + LGE imaging	11/177 (6.2%) with LV thrombus	All patients with LV thrombus were anticoagulated.
Meurin 2015 [17]	100 anterior STEMI with LVEF< 45%	Age: 59 ± 12 years Male: 71% DM: 20% Smoker: 43% LVEF: 33 ± 6%	CMR at a median of 30 days (range 20–40 days)	Cine + LGE imaging	26/100 (26%) with LV thrombus	All patients with LV thrombus were started on anticoagulation

*STEMI* ST-segment elevation myocardial infarction, *DM* diabetes mellitus, *MI* myocardial infarct, *CMR* cardiovascular magnetic resonance, *RCT* randomized controlled trial, *LGE* late gadolinium enhancement, *LV* left ventricular, *LVEF* left ventricular ejection fraction, *MACE* major adverse cardiovascular event, *CCF* congestive cardiac failure, *FFP* first pass perfusion

### Incidence of LV thrombus

**Fig. 2:**
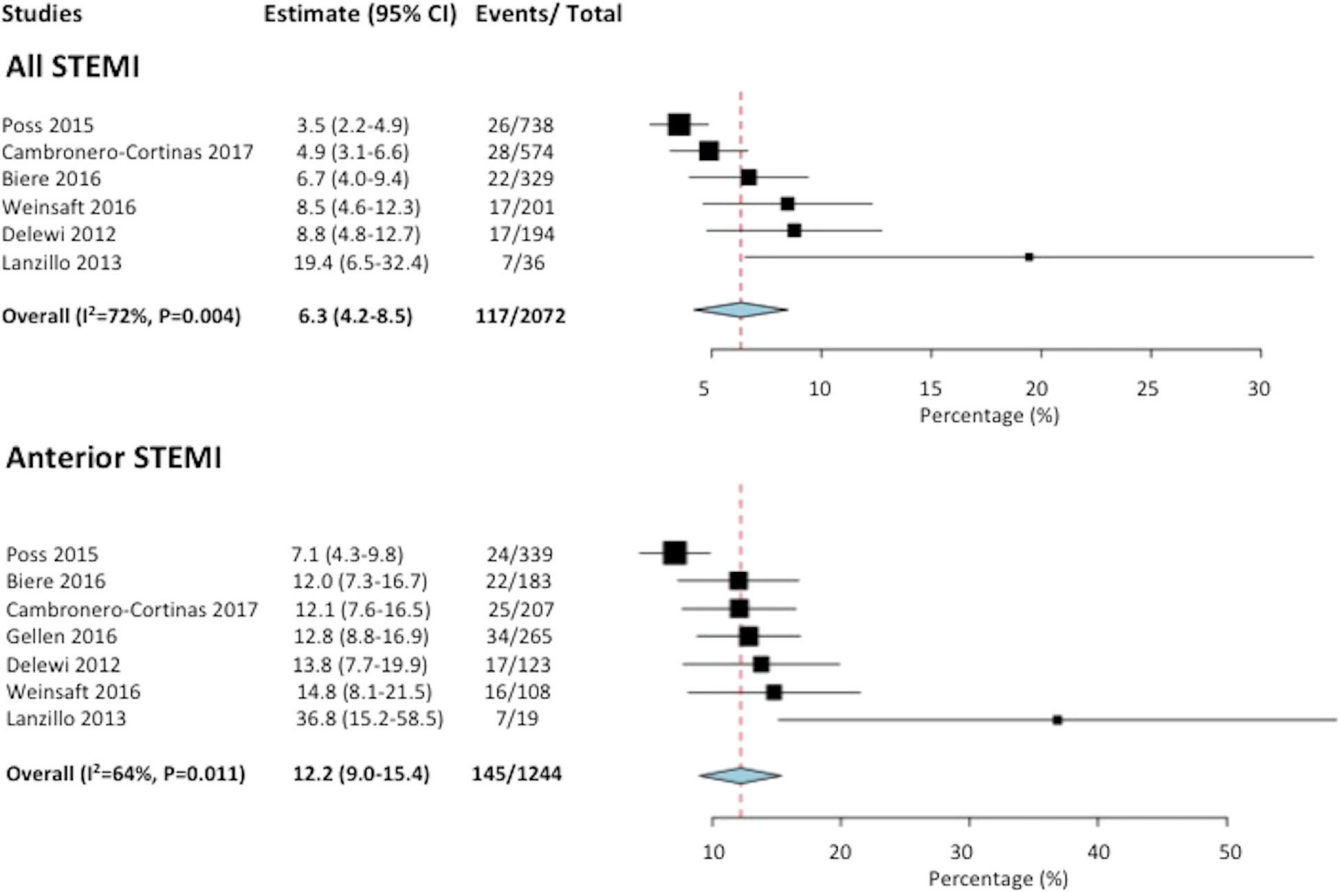
Forest plot of incidence of left ventricular (LV) thrombus by cardiovascular magnetic resonance (CMR) in ST elevation myocardial infarction (STEMI) patients treated by primary percutaneous coronary intervention (PCI). Forest plot showing the overall incidence of LV thrombus in STEMI and those with an anterior STEMI

Six studies [4, 10–14] included data on LV thrombus in anterior versus non-anterior STEMI. The overall incidence of LV thrombus in STEMI patients (*n* = 2072) was 6.3% (95%CI 4.2–8.5), I^2^ of 72% (Fig. 2) with 96% (111/116) of thrombi occurring with anterior STEMI. Among those with anterior STEMI only (7 studies, *n* = 1244) [4, 9–14], the incidence of LV thrombus was 12.2% (95%CI 9.0–15.4%), I^2^ = 64%, (Fig. 2).

Sensitivity analysis was performed by excluding the studies by Poss 2015 [10] and Lanzillo 2013 [11] (outliers on the Forest plot in Fig. 1). The overall incidence of LV thrombus was similar at 6.5% (95%CI 5.0–7.9%) STEMI (*n* = 1298) and was 12.8% (95%CI 10.6–15.0%) for anterior STEMI (*n* = 886), with I^2^ of 9% and 0%, respectively.

Three studies [15–17] included only patients with LVEF < 40–45%. One study [13] also reported the incidence of LV thrombus in those with anterior STEMI and LVEF< 50%. When only anterior STEMI with reduced LVEF were considered, the pooled incidence of LV thrombus in the 447 patients was 19.2% (95%CI 7.7–30.8%), I^2^ = 90%.

Three studies (acute CMR performed within the first week) also had a repeat CMR between 3 to 6 months [12–14]. LV thrombus resolved in 88% (50/57) of cases. However, late LV thrombus was detected in an additional 3.2% (95%CI 1.4–5.1%) of cases (29/868) on the 3 to 6 months CMR.

### Sensitivity and specificity of TTE against CMR

Three studies [4, 11, 12] presented data on LV thrombus detection by both TTE and CMR. Among the 431 patients with both CMR and TTE data, using CMR as the reference standard, the sensitivity of TTE was only 29% (95%CI 17–45%) with a specificity of 98% (95%CI 96–99%).

For anterior STEMI with reduced LVEF, 3 studies also presented data on the detection of LV thrombus by both TTE and CMR. For the 246 patients, the sensitivity of TTE for LV thrombus was 70% (95%CI 56–82%) with a specificity of 98% (95%CI 94–99%).

### Embolic and bleeding complications

Three studies [12, 13, 17] reported embolic and bleeding complications separately. All patients with LV thrombus were started on triple therapy (anticoagulation together with dual antiplatelet therapy).

After a follow-up period of between 1 and 2 years, the incidence of embolic complications (ischemic stroke and distal embolisation) was similar at 1.5% in both the LV thrombus group (1/68) on triple therapy (anticoagulation plus dual antiplatelet therapy) and no LV thrombus group (9/616) on dual antiplatelet therapy only, *P* = 0.25, I^2^ = 0%. However, bleeding complications were significantly higher in the LV thrombus group (6/68, 8.8%) than in the no LV thrombus group (3/616, 0.5%), *P* < 0.001, I^2^ = 65%.

## Discussion

**Fig. 3:**
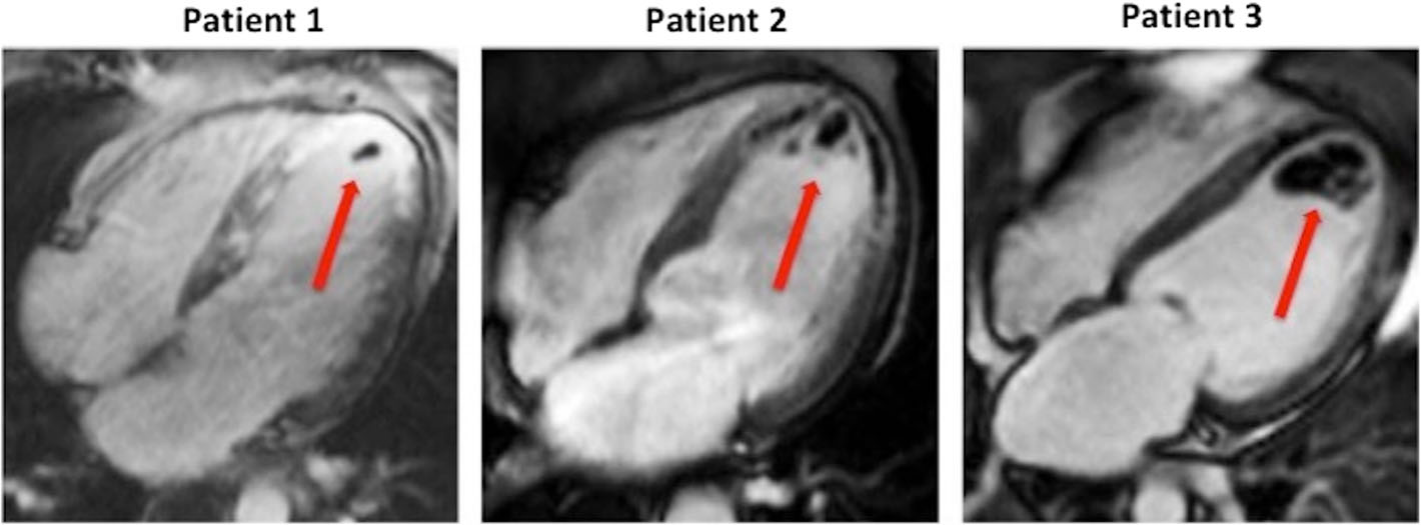
Early post-contrast CMR images of LV thrombus from 3 patients with acute STEMI treated by primary PCI. These 4-chamber views from 3 different patients illustrate LV thrombus of different sizes (red arrow) identified from the early post-contrast images acquired at high inversion time to null the avascular thrombus and MVO as black

In this meta-analysis of more than 2000 patients, overall CMR evidence of LV thrombus was > 6% of primary PCI STEMI patients. This is more than twice that reported by TTE [3]. We also confirmed that TTE had a poor sensitivity of only 29% when compared to CMR. The strength of CMR lies in its spatial resolution for morphological definition of the LV thrombus. Avascular thrombus can also be characterized and differentiated from neighbouring structures using gadolinium chelate contrast as illustrated in Fig. 3.

Expectedly, the large majority (96%) of LV thrombus by CMR occurred in those with anterior STEMI. The incidence of LV thrombus in anterior STEMI was more than 12%. In the subgroup of patients with anterior STEMI and LVEF< 50%, LV thrombus by CMR was detected in a remarkably high proportion of patients: one in five patients. Of note, the sensitivity of TTE was higher at 70% in this subgroup of patients.

The incidence of LV thrombus in the study Poss 2015 [10] was lowest (3.5%), despite including the largest number of patients (*n* = 738). This may be related to the early use of the potent glycoprotein IIb/IIIa inhibitor abciximab (intracoronary or intravenous) and warrants further investigation.

The incidence of LV thrombus by CMR is dependent on the timing of the CMR scan. An additional 3% of patients were identified to have late LV thrombus of the CMR performed between 3 and 6 months. Gellen et al. [9] recently showed that the highest LV thrombus detection rate (25%) was in those with CMR performed 9–12 days after anterior STEMI and Meurin et al. [17] showed a similar finding with those having the CMR scan between 8 and 15 days having the higher incidence of LV thrombus. These data suggest that the optimal timing of imaging for LV thrombus following an acute STEMI may be at 2 weeks.

Consistent with prior studies, risk factors for LV thrombus formation were anterior STEMI and LVEF< 50% [10, 13]. The presence of microvascular obstruction (MVO) [10] and apical wall motion abnormality [4] has also been associated with the development of LV thrombus. It has also recently been shown that LV thrombus was associated with a composite of heart failure, re-infarction and mortality [10]. This is likely a reflection of those with LV thrombus having larger infarct size, higher burden of MVO and lower LVEF.

Although the number of studies was limited, accounting for 684 patients, the incidence of embolic complications in those with LV thrombus and treated by anticoagulation was similar to those without LV thrombus. However, as expected, those with LV thrombus and triple therapy had higher incidence of bleeding complications. The large proportion of LV thrombus (88%) resolved on the repeat CMR performed between 3 to 6 months. A follow-up CMR scan as early as 3 months after STEMI may reduce the need for prolonged anticoagulation in a proportion of patients.

**Fig. 4:**
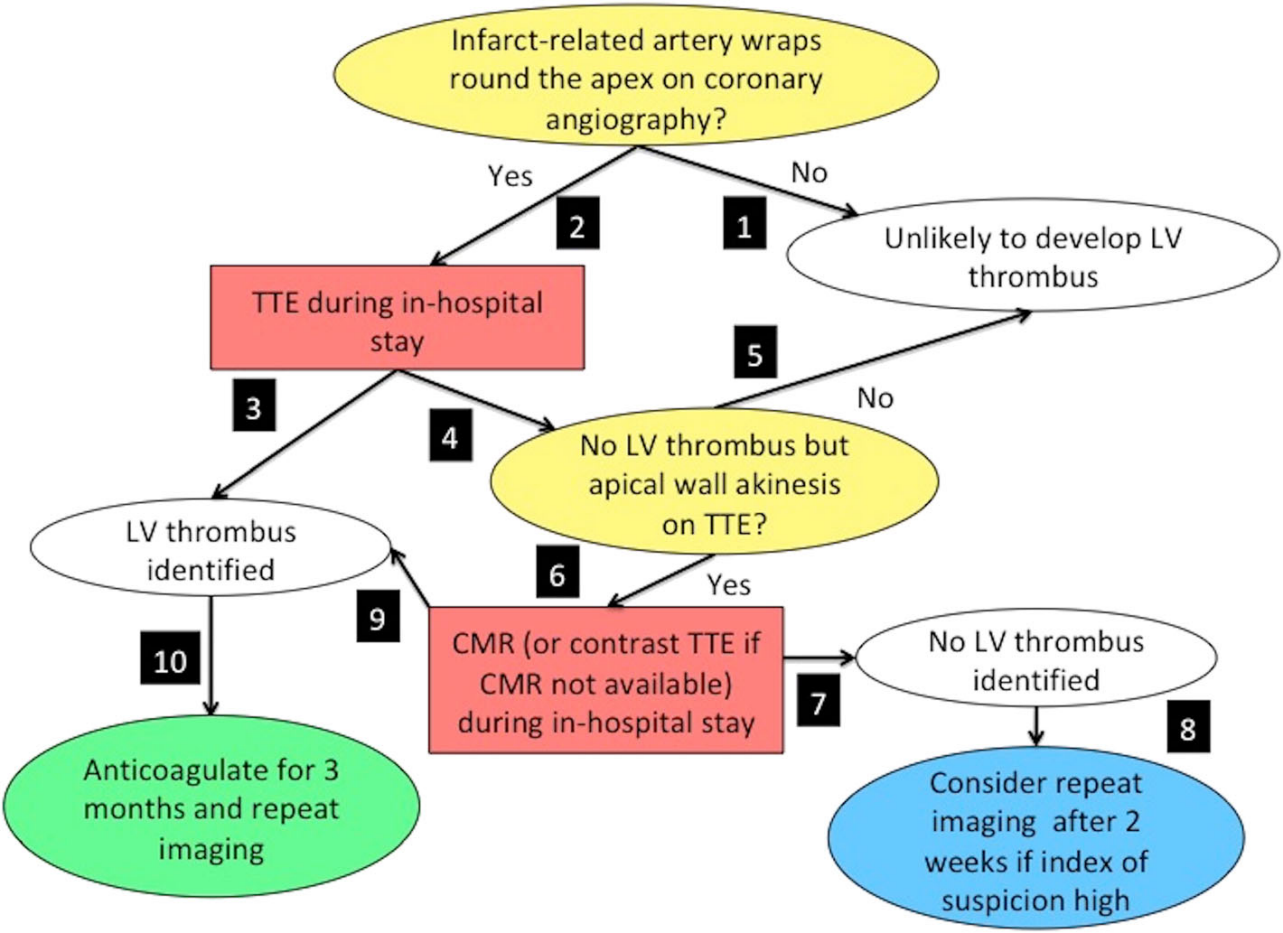
Algorithm for the detection of LV thrombus in reperfused STEMI patients in the clinical setting. This algorithm provides guidance on how STEMI patients at risk could be identified and how TTE and CMR could be integrated in the clinical setting to optimize the detection of LV thrombus

LV thrombus post acute STEMI is well recognised to occur predominantly at the apex [4] and in those with reduced LVEF [13]. The likely explanation why the incidence of LV thrombus is higher in anterior STEMI is likely because the left anterior descending artery subtends the largest amount of myocardium compared with the left circumflex coronary artery and right coronary artery and the left anterior descending artery wraps around the apex in three quarter of cases [18]. Moreover, a large anterior STEMI involving the apex would lead to blood stasis and endothelial injury, pre-requisites for the development of LV thrombus [19]. Despite the low sensitivity of TTE, it is more widely available and more affordable than CMR. Apical wall motion score has been shown to be useful to identify those would benefit from CMR imaging for LV thrombus detection. Therefore, we propose an algorithm, starting from the time of primary PCI that may be used to identify those most likely to develop LV thrombus and who may benefit from CMR if TTE is inconclusive (Fig. 4).

**Fig. 5:**
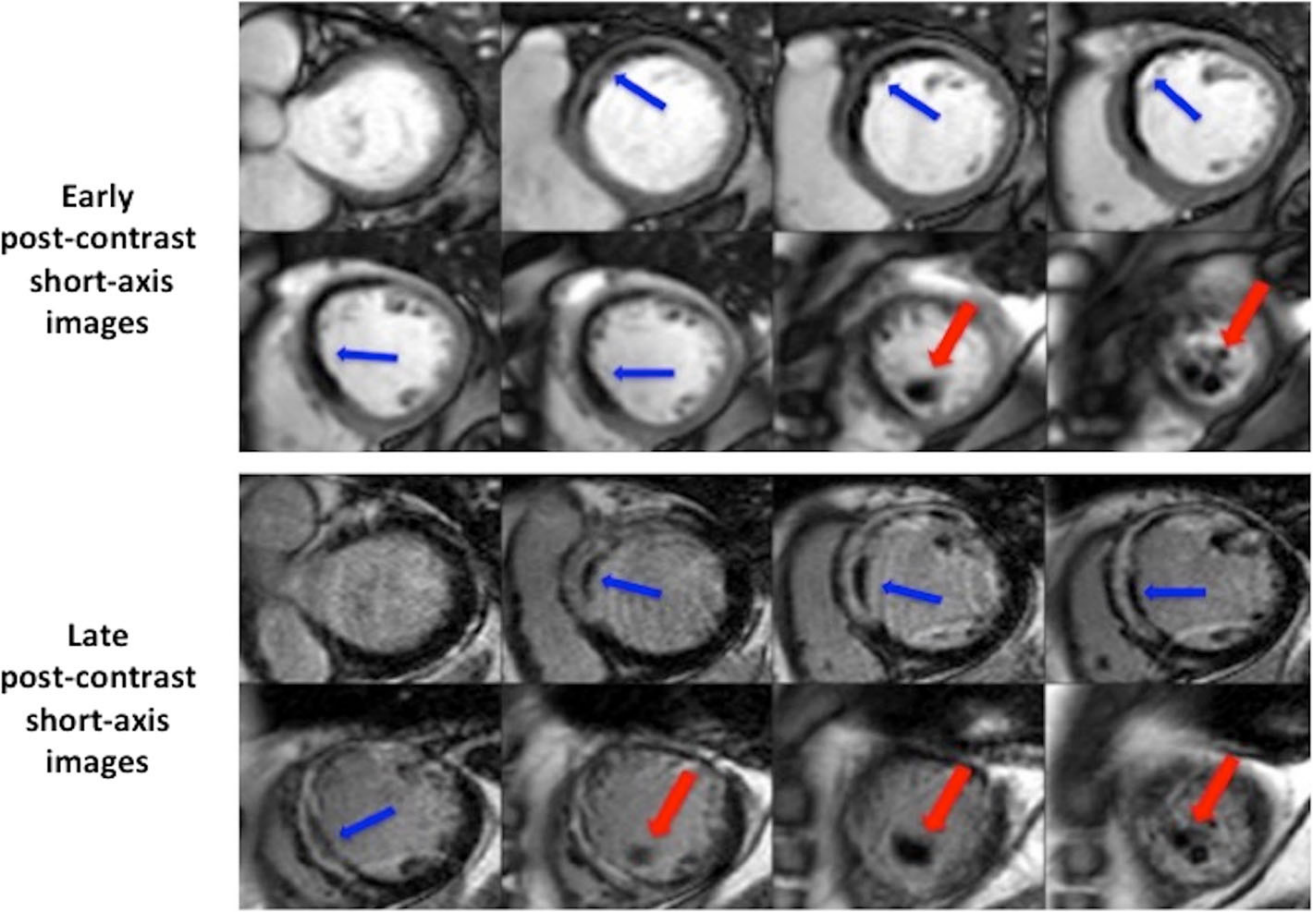
Differences in appearance between LV thrombus and microvascular obstruction (MVO). These are the early and late post-contrast short-axis images from a patient with LV thrombus and MVO. The red arrows show the LV thrombus and the blue arrows delineate the MVO

LV thrombus can sometimes be difficult to distinguish from MVO. The following previously described criteria [20] can be applied to differentiate between the two on late gadolinium enhanced images: location (LV thrombus tends to be intra-cavity whereas MVO is intra-myocardial); contrast fill-in on subsequent late gadolinium enhanced images would occur in the context of MVO but not with LV thrombus; differences in appearance (LV thrombus is usually well-defined with sharp borders whereas MVO tends to be patchy and inhomogeneous) as shown in Fig. 5.

Contrast TTE has also been used for the detection of LV thrombus with an improved sensitivity of 64% when compared to CMR [4]. This suggests that CMR may still be useful in high risk patients despite a negative contrast TTE.

In the chronic MI setting, in a cohort of patients with ischemic heart disease undergoing assessment for LV reconstruction surgery, the sensitivity of CMR against the pathological confirmation of LV thrombus was high at 88% [21]. The sensitivity of non-simultaneous TTE in that study was as low as 23% and that of intraoperative transesophageal echocardiography was only 40% [21]. The finding from our study is in line with this previous study showing a poor sensitivity of TTE to detect LV thrombus.

### Limitations

The techniques used in this meta-analysis to detect LV thrombus by TTE did not include echo contrast or newer 3D volumetric methods. The techniques used to detect LV thrombus by CMR (first-pass perfusion [14], conventional late gadolinium enhancement [9–13] or long inversion time gadolinium enhancement imaging [4]) and the timing of CMR in relation to the primary PCI were not standardized in the included studies. The studies were included in the forest plots showed high heterogeneity but we performed sensitivity analysis and this showed consistent results while improving the I^2^ significantly. Several of the secondary analyses included data from only 3 studies, but these constituted the largest pooled analyses and need to be confirmed in future larger prospective studies. Patient-level data were not available. Despite these limitations, our study provides a more accurate estimate of the true incidence of LV thrombus from a pooled analysis of > 2000 STEMI patients in the current primary PCI era. However, a proportion of STEMI patients with LV thrombus currently go undetected, as CMR is not part of routine practice. The natural history of these patients, especially in the current era of potent antiplatelet therapy is not known and could not be answered by this study. Lastly, CMR is currently not widely available, is relatively expensive and there may be an element of selection bias (only patients fit enough to lie flat, with no contraindications would tolerate the scan).

## Conclusion

CMR is a valuable tool for the detection of LV thrombus post-primary PCI for acute STEMI and the incidence remains clinically substantial at 6% of all-comers STEMI, 12% of anterior STEMI and 19% of anterior STEMI patients with reduced LVEF. We propose that CMR may have a role in high-risk STEMI patients (akinetic apex) and in patients with inconclusive TTE. Patients with LV thrombus treated with triple therapy (anticoagulation and dual antiplatelet therapy) have similar embolic complication rates but higher bleeding complication rates as compared with those with no LV thrombus treated with dual antiplatelet therapy only. We suggest a repeat CMR scan at 3 months post-STEMI to guide anticoagulation duration and to potentially reduce bleeding risk.

## Abbreviations

CI: Confidence interval
CMR: Cardiovascular magnetic resonance
EF: Ejection fraction
LV: Left ventricle/left ventricular
MVO: Microvascular obstruction
PCI: Percutaneous coronary intervention
PRISMA: Preferred Reporting Items for Systematic Reviews and Meta-Analyses
STEMI: ST-segment elevation myocardial infarction
TTE: Transthoracic echocardiography
